# Prediction of GCRV virus-host protein interactome based on structural motif-domain interactions

**DOI:** 10.1186/s12859-017-1500-8

**Published:** 2017-03-02

**Authors:** Aidi Zhang, Libo He, Yaping Wang

**Affiliations:** State Key Laboratory of Freshwater Ecology and Biotechnology, Institute of Hydrobiology, Chinese Academy of Sciences, Wuhan, 430072 China

**Keywords:** Hemorrhagic disease, Reovirus, Protein-protein interactions, Motif, Domain, GCRV

## Abstract

**Background:**

Grass carp hemorrhagic disease, caused by grass carp reovirus (GCRV), is the most fatal causative agent in grass carp aquaculture. Protein-protein interactions between virus and host are one avenue through which GCRV can trigger infection and induce disease. Experimental approaches for the detection of host-virus interactome have many inherent limitations, and studies on protein-protein interactions between GCRV and its host remain rare.

**Results:**

In this study, based on known motif-domain interaction information, we systematically predicted the GCRV virus-host protein interactome by using motif-domain interaction pair searching strategy. These proteins derived from different domain families and were predicted to interact with different motif patterns in GCRV. JAM-A protein was successfully predicted to interact with motifs of GCRV Sigma1-like protein, and shared the similar binding mode compared with orthoreovirus. Differentially expressed genes during GCRV infection process were extracted and mapped to our predicted interactome, the overlapped genes displayed different tissue expression distributions on the whole, the overall expression level in intestinal is higher than that of other three tissues, which may suggest that the functions of these genes are more active in intestinal. Function annotation and pathway enrichment analysis revealed that the host targets were largely involved in signaling pathway and immune pathway, such as interferon-gamma signaling pathway, VEGF signaling pathway, EGF receptor signaling pathway, B cell activation, and T cell activation.

**Conclusions:**

Although the predicted PPIs may contain some false positives due to limited data resource and poor research background in non-model species, the computational method still provide reasonable amount of interactions, which can be further validated by high throughput experiments. The findings of this work will contribute to the development of system biology for GCRV infectious diseases, and help guide the identification of novel receptors of GCRV in its host.

**Electronic supplementary material:**

The online version of this article (doi:10.1186/s12859-017-1500-8) contains supplementary material, which is available to authorized users.

## Background

Grass carp (*Ctenopharyngodon idellus*) is an important aquaculture fish widely cultured in Asian countries, especially in China. However, disease outbreaks in this species are very frequent and leading to huge economic losses. Grass carp hemorrhagic disease that caused by grass carp reovirus (GCRV) is one of the most serious diseases, which mainly outbreak in young fingerling and yearling fish [1]. GCRV is a double-stranded RNA virus that belongs to the *Aquareovirus* genus in the *Reoviridae* family. The genome of GCRV consists of 11 segments of dsRNA, and encodes eleven proteins, including seven structural proteins and four non-structural proteins [2, 3]. To date, a number of various GCRV strains have been isolated from diseased grass carp around the world. Based on difference in genome constitution, GCRV could be mainly clustered into three subtypes, the representative strains of three subtypes are GCRV-873 (subtype I), GD108 (subtype II), and GCRV104 (subtype III), respectively [2–5]. Identities of amino acid sequences among each two subtypes are less than 30% due to fast evolution [2, 3, 5]. GCRV subtype II, represented by GD108, named ‘Grass carp reovirus Guangdong 108 strain’, was isolated recently from diseased grass carp in China [2], its genome shows distinct molecular properties compared with other two reported subtypes GCRV strains [3]. In addition, GCRV subtype II is considered to be the most pathogenic and prevalent subtype in China. Phylogenetic analysis showed that GD108 may be closer to *Orthoreoviruse* than any other known species of *Aquareovirus* [3, 6]. The characteristics of GD108 proteins are listed in Table 1.

**Table 1:** Characteristics of GD108 proteins and the corresponding number of host proteins targeted by motifs

Genome segment	Segment name	Uniprot accession no.	Genome length (bp)	Protein length (aa)	Number of PPI	Predicted function
L1	VP1	E7DDK3	3,928	1,294	910	Guanylyl transferase/Capping Enzyme
L2	VP2	E7DDK4	3,867	1,273	949	RNA-dependent RNA polymerase
L3	VP3	E7DDK5	3,752	1,232	636	NTPase/helicase
M4	NS1	E7DDK6	2,263	716	910	Non-structural, possibly involved in the formation of viral inclusion body
M5	VP5	E7DDK7	2,230	726	765	Inner capsid protein
M6	VP4	E7DDL3	2,028	650	1083	Major outer capsid protein
S7	Sigma1-like protein	E7DDK8	1,604	512	854	Minor capsid cell attachment protein, possibly
S8	Unknown	E7DDK9	1,563	361	16	Major inner capsid protein
S9	VP6	E7DDL0	1,320	418	435	Non-structural, possibly invplved in the formation of viral inclusion bodies
S10	Sigma NS-like protein	E7BY76	1,124	354	573	Non-structural, possibly
S11	Unknown	E7DDL2	1,027	310	389	Non-structural protein

The seventh column represents the number of host proteins whose domains were predicted to interact with motifs of the corresponding virus protein

Previous studies of grass carp hemorrhagic disease mostly focused on functions of limited genes, especially immune associated genes, such as pattern recognition receptors, *TLR2, TLR3, TLR4* and so on [7]. However, the pathogenesis process of GCRV infection remains largely unknown. Viruses are referred to as obligate parasites, they cannot reproduce outside their hosts, hence need to tune host cellular machinery by interactions between viral and several host proteins during viral infection [8]. Therefore, virus-host protein-protein interactions (PPIs) play a crucial role in the outcome of infection and establishment of disease. Studying PPIs may help us understand the possible roles of viral proteins. Until now, viral-host PPIs have been keenly studied by employing both computational and experimental approaches [9–11]. Compared with within-host PPIs interfaces, virus-host PPIs interfaces tend to be more transient, targeted by more host proteins, more regulatory in function, faster evolving, and rely more on convergent evolution to achieve interface mimicry [8]. Hence, experimental methods in identifying virus targeted proteins are challenging and costly. Until now, many computational methods have been widely used in genome-wide mapping of pathogen-host PPIs for selected pathogens [12–16]. Viruses have few domains and their structures are hard to find by comparative modeling, thus traditional methods (homology-based, structure-based) could not work in virus-host PPIs system. Recently, the potential functional roles of interactions mediated by motifs and their counterpart domains in viral infection have been addressed in a number of recent articles [13, 16], demonstrating the power of motif-based approach. For GCRV and its host grass carp, heretofore, there are few published reports about their PPIs, only the PPIs targeted by VP7 protein in GCRV GD873 were screened by using yeast two-hybrid system [17]. Hence there is an urgent need to study the GCRV virus-host interactome systematically, which may help us to understand the underlying pathogenesis of GCRV infection.

In the present study, we predicted the GCRV virus-host PPIs on a genome scale by using GD108 as the representative strain. We focused on PPIs mediated by relationships between short motifs on GCRV proteins and grass carp protein counterpart domains that known to interact with those structural motifs. We further explored the characteristics of the PPIs network, and found one PPI between Sigma1-like protein in GCRV GD108 and host protein junctional adhesion molecule A (JAM-A), the orthologous gene of JAM-A in human has been proved to be the only known receptor for mammalian reovirus (MRV). We further evaluated the influences of the interactions by analyzing expression data during different infection stages. Finally, functional annotations and pathway analysis were performed to explore the potential mechanisms associated with host targets. The present work provided the first system-based framework about the interactome of GCRV infection, the findings may complement and guide further experiments aiming to identify host hub genes that are necessary for GCRV survival and replication within the host cells.

## Results

### Overview of pipeline for constructing GCRV virus-host interactome

PPI is always driven by contact of essential residues around interface in DDIs (domain-domain interactions) and DMIs (domain-motif interactions) [11]. Compared with within-host PPI interfaces, virus-host PPI interfaces tend to be more transient and targeted by more host proteins. Since virus proteins always do not have known domains due to fast evolutionary rate [8, 18], it is hard to predict virus-host PPIs relying on DDIs-based and homology-based strategies. Thus we only explored the DMIs information to construct virus-host PPIs network.

Firstly, we performed domain annotation against the grass carp protein sequences by the software InterProScan [19], here we used PfamA as the domain reference database. Secondly, structural descriptors for motif-domain interactions were collected from two databases, 3did and iELM, respectively [20, 21]. Thirdly, motif pattern searching was performed against GCRV GD108 protein sequences. Early study demonstrated that the structural information of the motifs are strongly related to PPIs, and sequences exposed on the surface should be more accessible than those that are buried [22]. Hence, we took surface accessibility of these motif residues into account to reduce the rate of false-positives by using the NetSurfP package [23]. More than half of exposed residues in a motif is used as the cutoff to select reliable motifs, this threshold was also used in previous work [16]. Finally, for the two resulting datasets, a stringent criterion was used to filter a reliable virus-host PPIs. If one GCRV protein was both predicted to interact with the same domain in the two datasets, we considered that this interaction pair was true. However, each database has its specific domains, which may involve in important interaction events. For example, v-set domain (PF07686) was only included in 3did database, whereas Pkinase domain (PF00069) was only included in iELM database. Hence, for both databases, we collected the interactions between database-specific domains and motifs. The occurrences of motif patterns were evaluated, and only rarely appeared motifs were reserved and added to the previous interaction pairs.

3did database includes 549 motif patterns, 149 domains, and 651 domain-motif interactions, including inter-chain interactions and intra-chain interactions. However, iELM database includes 103 domains, 206 motif patterns, and 225 domain-motif interactions. There are only 48 domains appeared in both databases, suggesting the discrepancy in database construction. These overlapped domains are more likely to interact with motifs, such as SH3_1, PDZ, WW domains, which were once proved to be the most highly interactive domains, recognized by short peptides, in signaling pathways [18]. The database-specific domains were reserved only when its corresponding motif patterns appeared sporadically (occurrences < =4). Detailed information of identified motifs based on two databases was listed in Additional file 1 and Additional file 2, respectively.

**Fig. 1:**
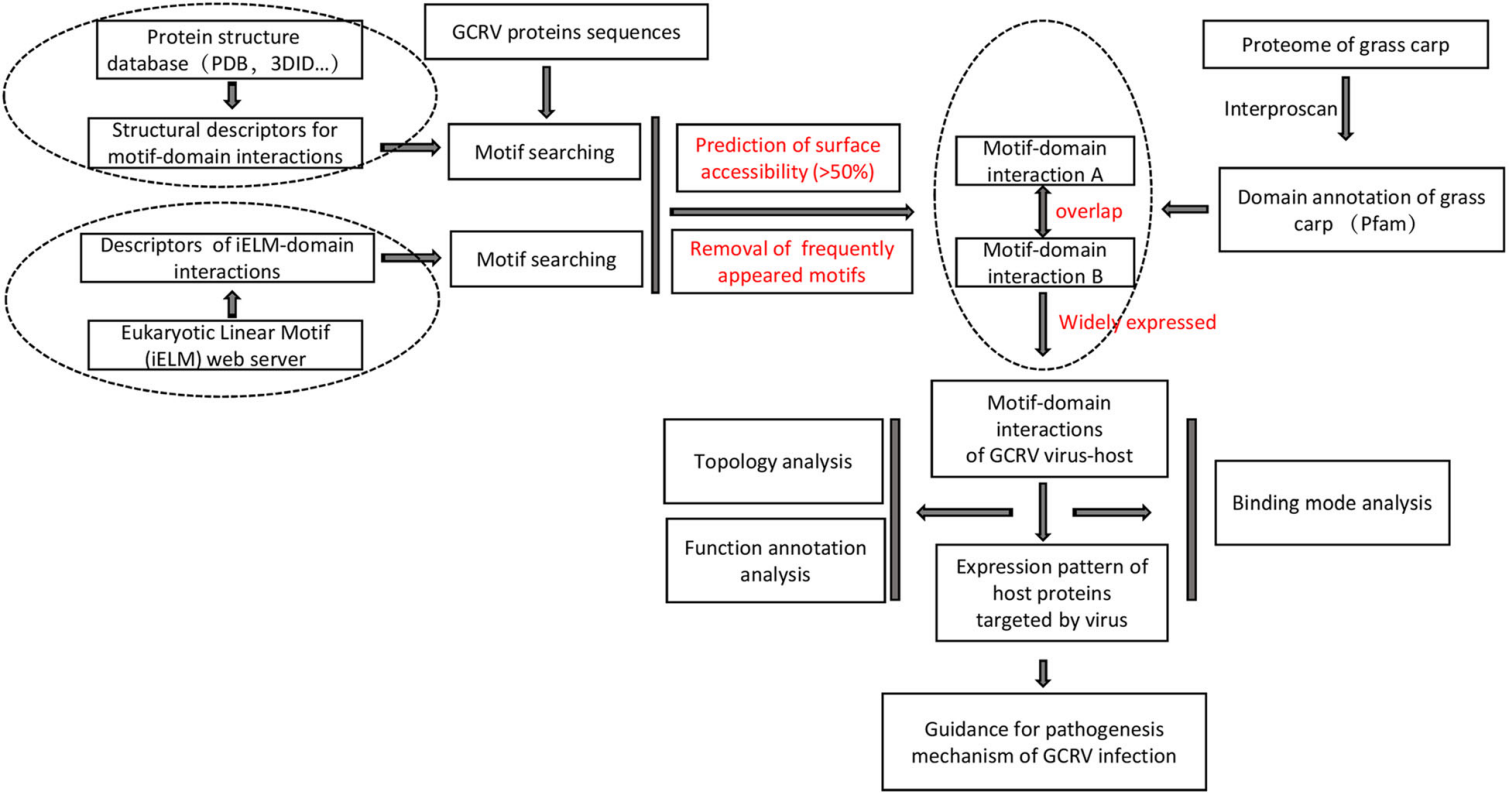
Pipeline for the prediction of GCRV virus-host protein interactome based on structural motif-domain interactions

**Fig. 2:**
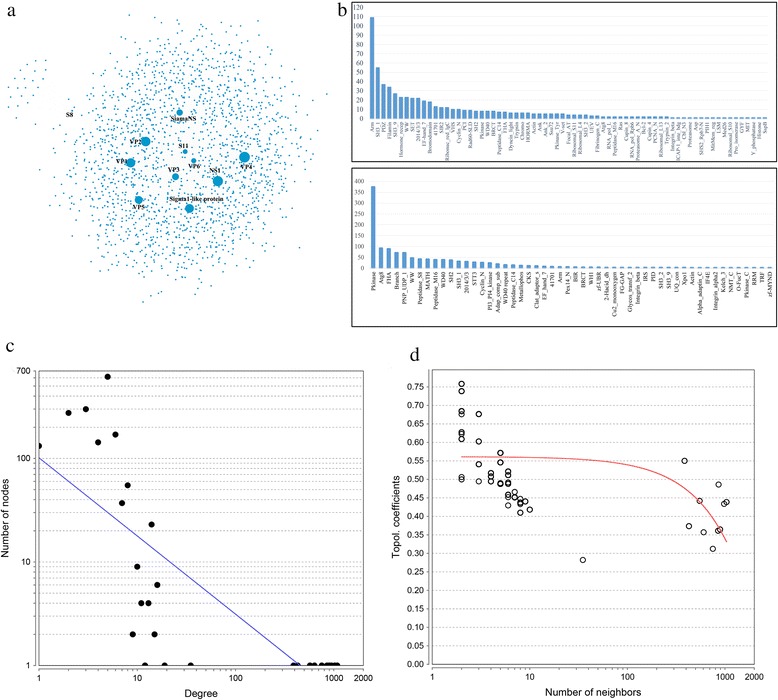
Characteristics of GCRV virus-host protein interaction network. **a** GCRV virus-host protein interaction network, the node represents proteins, the line links the nodes, is colored as light grey. The node size is proportional to the number of degree. **b** Frequencies distribution of domains targeted by virus GCRV proteins. The y axis represents number of frequency of domain, and the x axis represents domain name. The above one depicts the distribution of domains that predicted based on 3did database, and the below one depicts the distribution of domains that predicted based on iELM database. **c** Node degree distribution of predicted GCRV-host PPIs network. The x axis represents number of degree, and the y axis represents number of node. **d** Topological coefficients of predicted GCRV-host PPIs. The x axis represents number of neighbors, and the y axis represents topological coefficients

Thereafter, we linked the motifs to host proteins containing its domain partners. Previous studies proved that host proteins in virus-host PPIs expressed abundantly across multiple tissues [24]. Thus we filtered out proteins that only expressed in less than four tissues using RNA-seq data from our previous work [25]. The workflow is shown in Fig. 1. We performed motif searching against 11 proteins in GCRV GD108 independently. About 20% of motifs were filtered out due to ‘buried’ property. Eventually, we obtained an interaction database, including virus proteins, motifs of virus proteins, host proteins, domains of host proteins. The GCRV virus-host interaction network, in csv format, was generated (Additional file 3), its visualization can be explored interactively using the freely available Cytoscape software [26], which was shown in Fig. 2a.

### Characteristics of GCRV virus-host interaction network

Various types of motif patterns were detected among different proteins. We found that several virus proteins shared the same interacting domains by using different motifs in both databases, such as ARM, SH3-1, PDZ domains. In contrast, SspB domain was only predicted to interact with S8 protein. We also found that the number of occurrences differs greatly across domains, as shown in Fig. 2b, Arm and Pkinase domain has the highest occurrence. Interestingly, we found that a lot of motifs based on one database was also identified in the other database. For example, for Sigma1-like protein, one motif ‘VTSLD’ (motif pattern: [VT..D]) was identified based on 3did database, meanwhile, one motif ‘AVTSLDA’ (motif pattern: [..(T)..[DE].]) was also identified based on iELM database. Both of them are located in the same position of about 55 bp and predicted to interact with FHA domain. This phenomenon demonstrates the reliability of database-combined strategy.

GCRV virus-host interaction network consists of 11 virus proteins and 1757 host proteins. The statistics information of GD108 and the number of predicted host proteins were listed in Table 1. The total number of host protein is reasonable, for Hepatitis C virus, there are more than 1730 host proteins reported from previous studies [27]. Additionally, for well-studied HIV virus, the number of host-virus PPIs reaches up to 2431 [27]. However, it seems that the average number of interactors for one virus protein is a little higher, which results from that a lot of host targets were shared by several virus proteins. By analyzing the network, we found that the degrees of this network followed the power-law distribution (Fig. 2c), suggesting that most proteins are involved only a few PPIs while only a small number of proteins participate in a large number of PPIs. The topological coefficients was plotted to estimate the tendency of the nodes in the network to have shared neighbors, which was shown in Fig. 2d.

### Binding mode analysis of Sigma1-like protein with the JAM-A protein

For GCRV, the outer capsid proteins, such as VP7, VP5, are always proved to play key roles in virus’s attachment and infection by interacting with proteins expressed on host cell surface [28], but their receptors remain unknown. Until now, only one gene, named junctional adhesion molecule A (JAM-A) in human, was known as the primary receptor for MRV by interacting with Sigma1 protein [29–31]. MRV utilizes Sigma1 protein as attachment molecular to interact with JAM-A during epithelial tight junction formation, and infection occurs through bloodstream dissemination from the intestine to sites of secondary infection [32]. Likewise, JAM-A in grass carp was also assumed to be the most probable receptor for GCRV by our previous work [33]. However, which virus protein that JAM-A might interact with remains unknown. In our study, the resulting GCRV virus-host interactome may provide some hints of this question. We found that several virus proteins were predicted to interact with the V-set domain of JAM-A by using different motif patterns, especially S10 segment named Sigma1-like protein, the pattern of “D.[AGS][FL]” occurs three times around the position of about 300 bp in Sigma1-like protein (Table 2).

**Table 2:** Diverse motifs of the GCRV proteins predicted to interact with grass carp *JAM-A* protein

Virus protein name	Domain	Motif pattern	Motif	Motif_start	Motif_end	Motif_length	Surface accessibility
Sigma1-like	V-set	Y..S…D	YVGSSSVD	190	198	8	EBBEEEBE
Sigma1-like	V-set	D.[AGS][FL]	DLGL DGGL DLSL	297 341 350	354 345 354	4 4 4	BEEE EEBE EEEE
VP3	V-set	N.NG	NTNG	1159	1163	4	EEEB
VP4	V-set	N.NG	NING	11	15	4	BBEE
VP4	V-set	N.NG	NPNG	41	45	4	BEEB
VP4	V-set	N.N.S.H	NPNDSAH	532	539	7	EEEEEEE

*E* exposed residue, and *B* buried residue

**Fig. 3:**
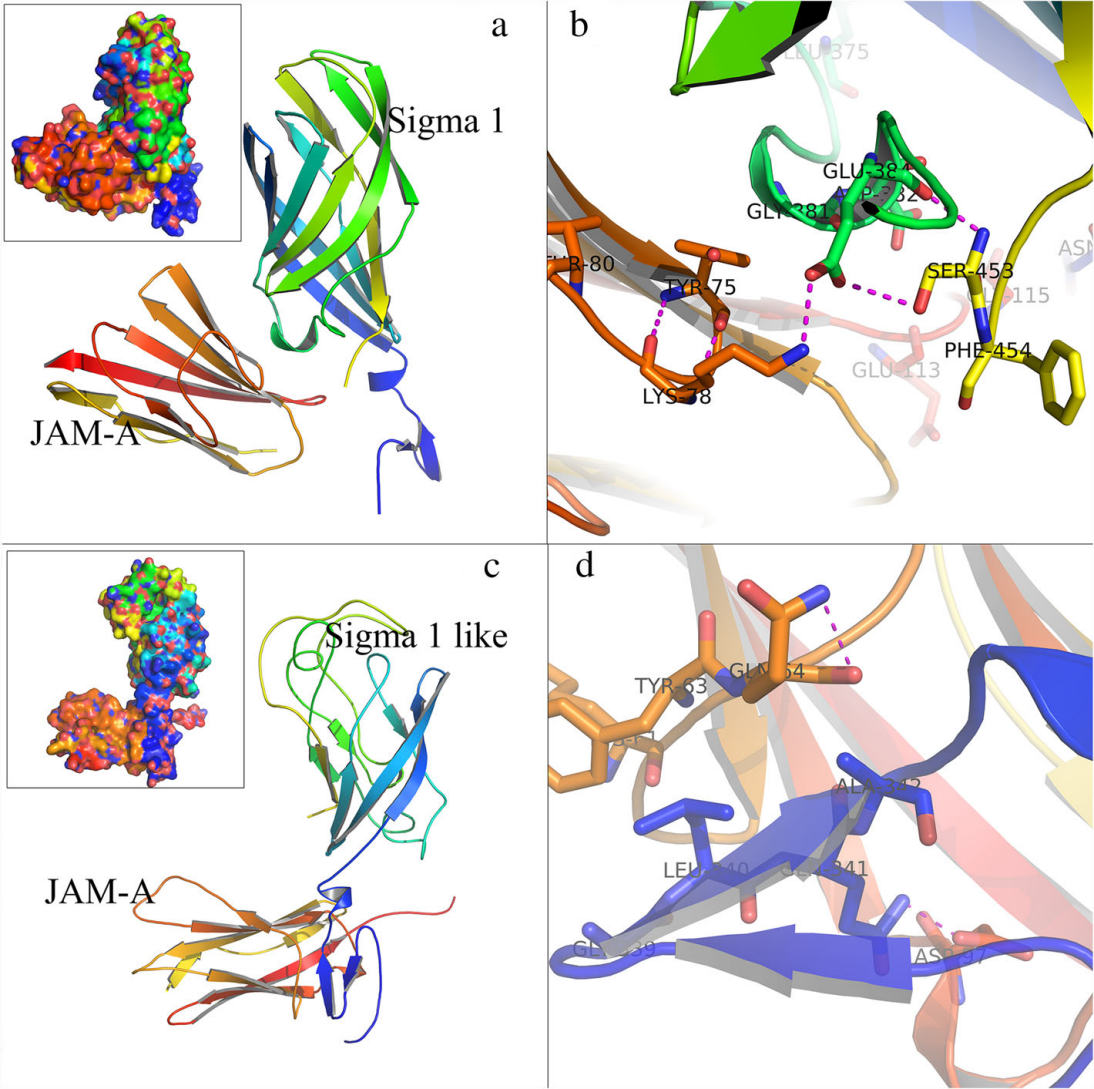
Structural comparison between Sigma1 proteins in complex with their receptors JAM-A proteins. **a** Complex structure of MRV Sigma1 protein and human JAM-A protein (PDB ID: 3eoy). **b** Binding mode between MRV Sigma1 protein and human JAM-A protein. **c** Predicted structures of GD108 Sigma1-like protein and grass carp JAM-A protein using the I-TASSER server [51]. Proteins docking was carried out by using the Zdock server [52]. **d** Binding mode between GD108 Sigma1-like protein and grass carp JAM-A protein

**Fig. 4:**
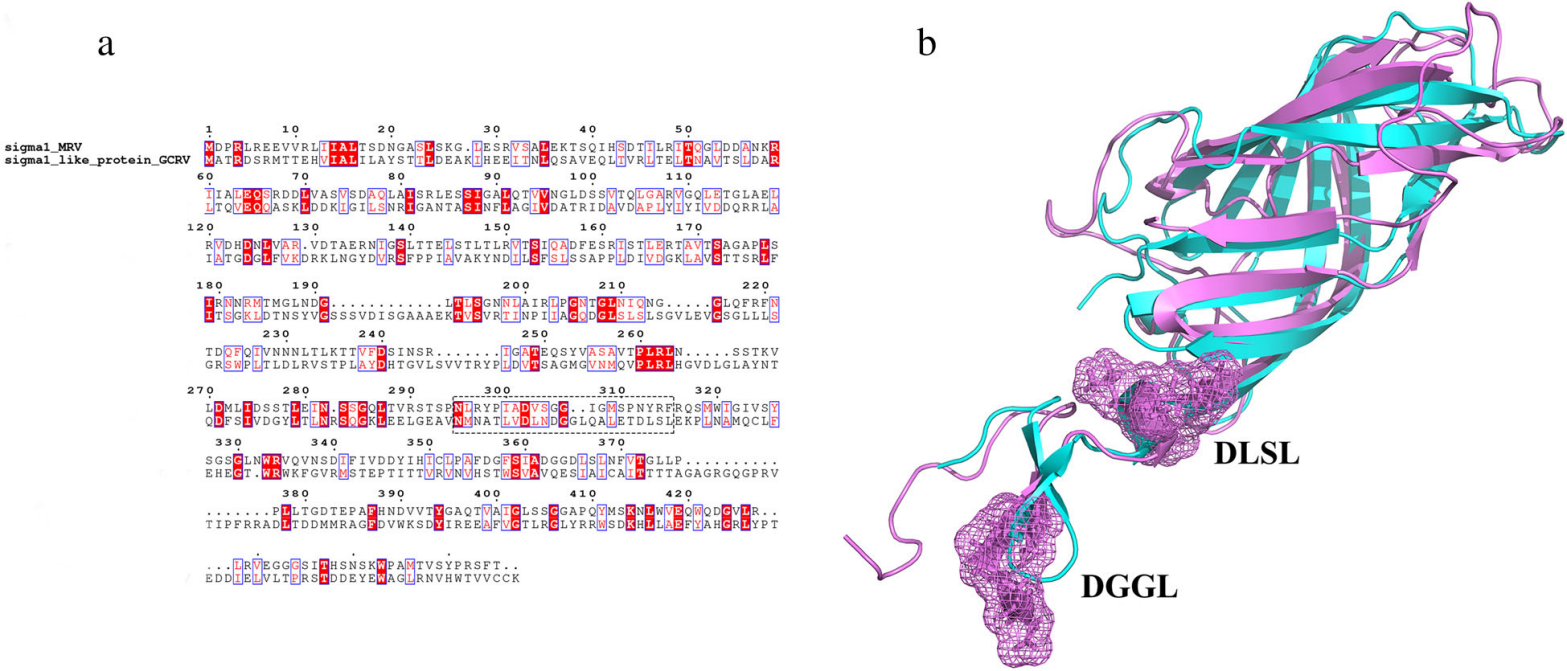
Sequence and structure alignments between Sigma1 and Sigma1-like protein. **a** Sequence alignment between Sigma1 and Sigma1-like protein. The motifs around the predicted interface between Sigma1-like protein and JAM-A are labeled with a dotted box. **b** Structure alignment between Sigma1 and Sigma1-like protein. Sigma1 protein was light blue colored, and Sigma1-like protein was purple colored. The motifs around the predicted interface between Sigma1-like protein and JAM-A are labeled with mesh surface

We performed structure comparison aiming to illuminate the underlying interaction mode between JAM-A and Sigma1 proteins. For MRV, Sigma1 protein is a fibrous trimer, consisting of an elongated tail N-terminal domain and a globular head C-terminal domain. The N-terminal domain inserts into the virion, whereas the C-terminal domain projects away from the virion surface. Hence, Sigma1 protein attaches the virion to the host cell membrane [34, 35]. We found that the predicted model of Sigma1-like protein have the similar three-dimensional structure as Sigma1 protein. Both Sigma1 proteins contain one C-terminal globular head domain with a compact stranded-barrel, and an elongated tail N-terminal domain. But Sigma1-like protein possesses more loops and shorter β pleated sheet. We set the predicted motif residues as binding sites for docking, the docking results additionally revealed that these two Sigma1 proteins share the similar binding mode with JAM-A proteins (Fig. 3a, c). Both of them bind the V-set domain of JAM-A by using the linker region at the bottom of the C-terminal globular head domain. The interactions involved extensive ionic and hydrophobic contacts (Fig. 3b, d). Sequence and structure alignments between Sigma1 and Sigma1-like protein further supported the above viewpoint (Fig. 4). Although alignment result showed obvious widespread discrepancy in amino acid sites, there are still quite a lot conserved residues. In addition, the region of our predicted motif residues is also conserved, this might be due to the function constraint of acting as binding sites with JAM-A protein. Based on these results, it seems reasonable to suppose that Sigma1-like protein adopt the same strategy of MRV Sigma1 to mediates attachment to cell-surface receptors. Moreover, the evolution of GD108 and its relationships to MRV and GCRV appears to be much more interesting in terms of its unique presence of an outer-fiber protein (Sigma1-like protein) as compared with other types of GCRV.

### Expression pattern of putative host proteins targeted by GCRV

**Fig. 5:**
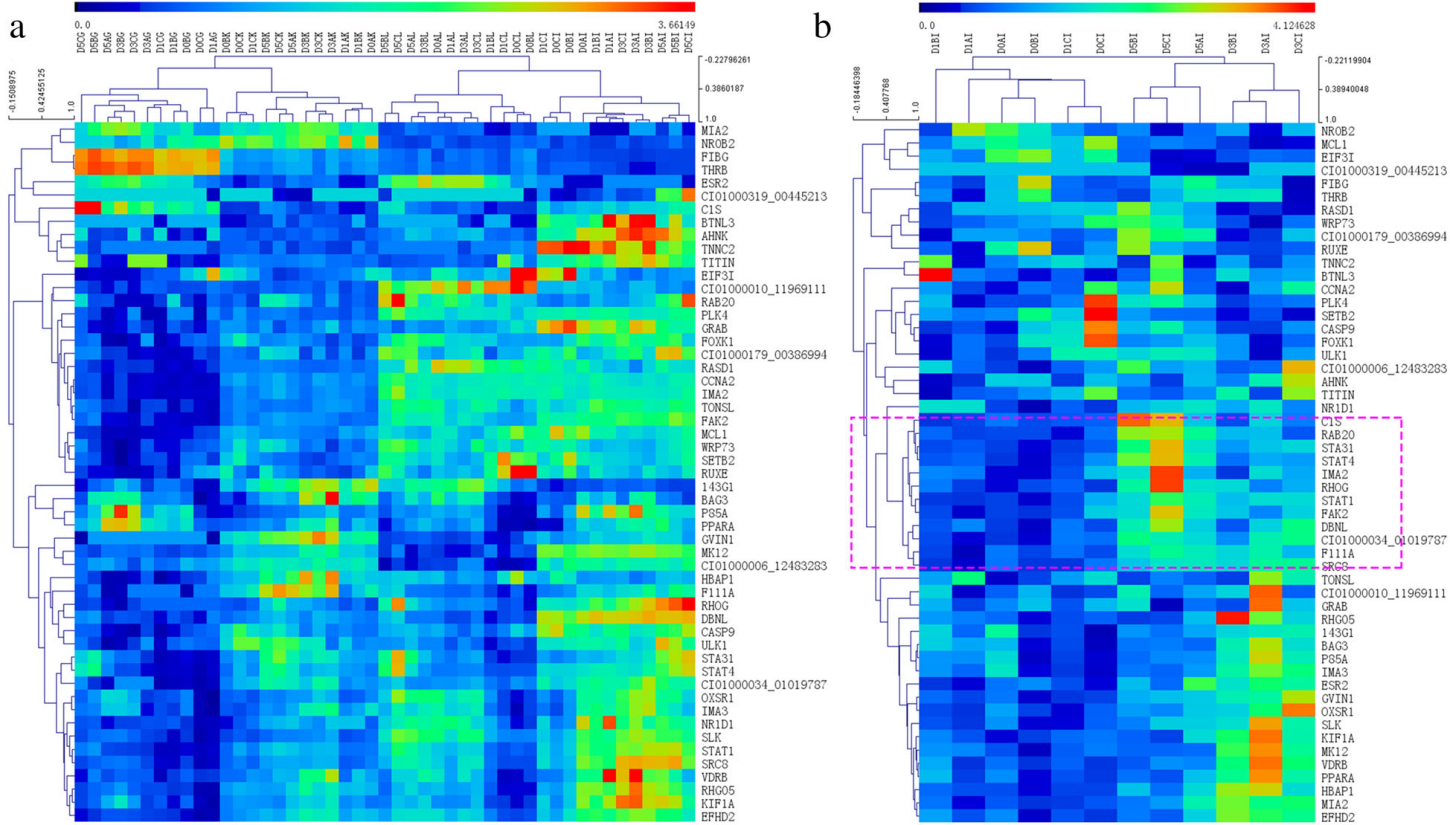
Expression patterns of DEGs that targeted by GCRV. **a** Hierarchical cluster analysis of significant DEGs expression profiles from four tissues with three replicates at the four time points after (0 h, 1 h, 3 h, 5 h) GCRV challenge. **b** Hierarchical cluster analysis of significant DEGs expression profile of intestine. The color is proportional to the expression level, which was subjected to log2 ratios transformation. The x axis represents different samples, and the y axis represents gene symbols. We named one sample by four letters, the first letter ‘D’ means ‘DEGs’, the second letter means the replicate Number (A, B, C), the third letter means the time point (0 h, 1 h, 3 h, 5 h), the fourth means the tissue, G, I, L, K represent gill, intestine, liver, head kidney independently. Take ‘D5CI’ for example, it means DEGs from the third replicate sample of intestine after 5 h GCRV challenge

When a pathogen infects its host, extensive PPIs happen along with related altered gene expression level. Thus, transcriptomic signatures may be useful in identifying genes that play crucial roles during infection process. We obtained RNA-seq data from four diseased grass carp tissues (gill, intestine, liver, head kidney) with three replicates at four times after (0 h, 1 h, 3 h, 5 h) GCRV challenge [36], and investigated the expression pattern of host proteins targeted by virus during various stages of GCRV infection. We identified DEGs compared with the profile of 0 h time point independently. The four resulting DEGs sets were merged together, and mapped to our predicted host targets. Hence, the overlapping genes were not only host targets, but also demonstrating different expression. A total of 53 DEGs (*p*-value < 0.05, |log2 (Fold_Change)| > 1.5) were present in our virus-host interactome. We listed the detailed information about these genes in Additional file 4. The results showed that these genes displayed different tissue expression distributions among different tissues (Fig. 5a). The global expression level in intestinal is higher than that of other three tissues, which may suggest that the functions of these genes are more active in intestinal. This phenomenon of expression pattern is consist with previous assumption that GCRV infection among grass carp population are mainly mediated by food intaking from intestinal digestion to other tissues. These DEGs were clustered into three clades by using hierarchical cluster method, genes of one clade showed the similar expression trend and may function together in the same pathway. Take the intestinal expression profile for example (Fig. 5b), genes inside the red dotted box, including *CIS, STA31, STAT4, STAT1* and so on, are mainly involved in negative regulation of cytokines, and function in signaling through the JAK/STAT pathway. Likewise, *RHOG, RAB20, DBNL, CTTN* and *FAM111A* genes participate in pathways of micropinocytosis and phagosomes. *FAM111A* gene is proved to be targeted by virus to overcome host range restriction to promote virus DNA synthesis and play a key role in defense response to virus [37]. Hence, those genes were not only predicted to interact with virus proteins, but also demonstrated apparent altered expression level, may play important roles during the pathogen invasion process, and are worthy of further studies. Moreover, the different profiles in other tissues indicated the different underlying pathogenic pathway.

### GO annotation and pathway enrichment analysis of putative host proteins targeted by GCRV

**Fig. 6:**
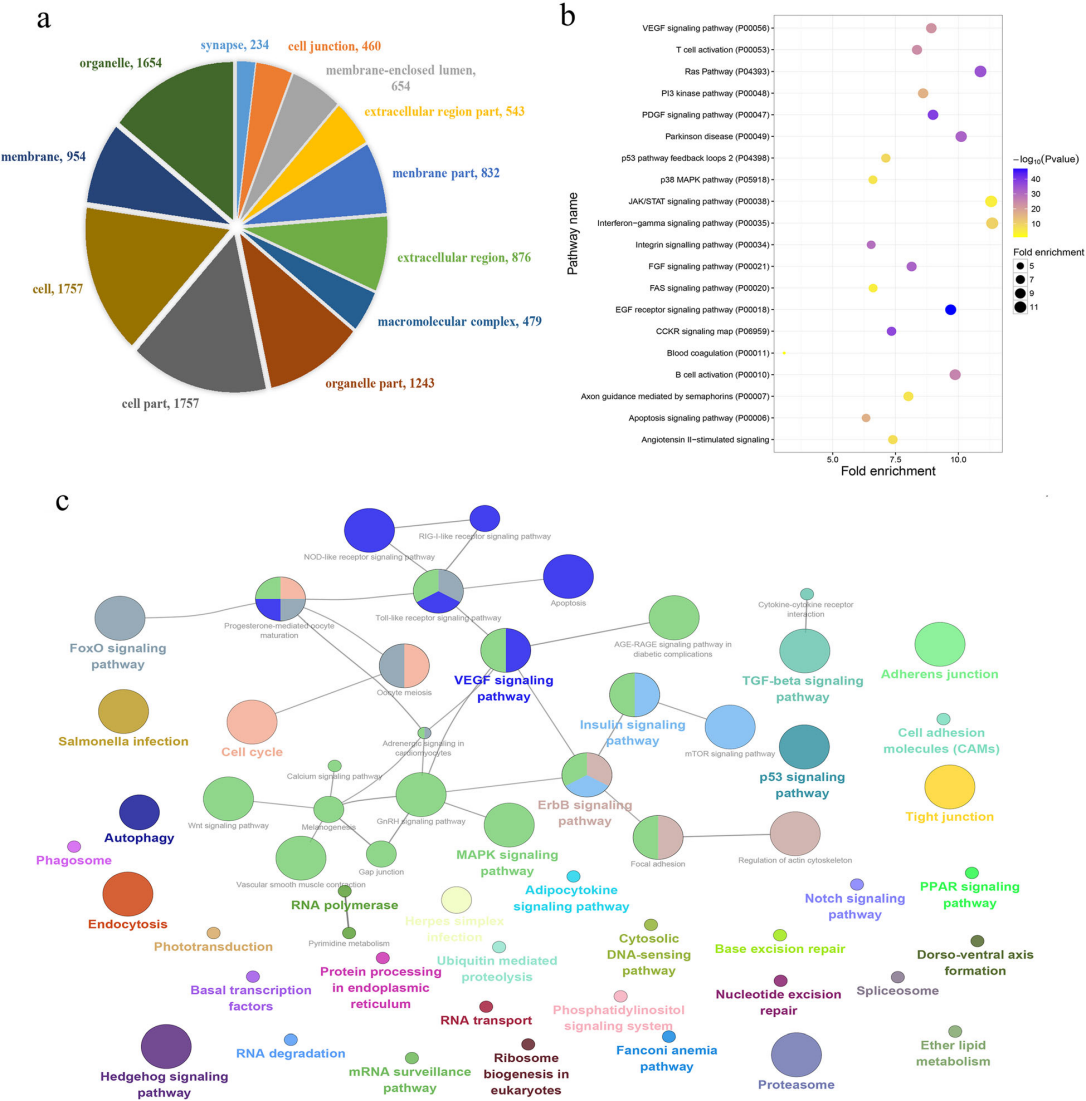
Function enrichment analysis of predicted host proteins targeted by GCRV. **a** Cellular component annotation, numbers behind the terms represent the gene numbers. **b** Pathway annotation using PANTHER overrepresentation test (*p*-value < 0.05), Bonferroni correction was adopted. The size of circle is proportion to estimated fold enrichment, and the color is proportion to -log10 ratio transformation of *p*-value. **c** Gene ontology significant enrichment analysis of host proteins targeted by GCRV. The graph was generated using ClueGO program [57]. Detailed information of GO terms was listed in Additional file 5

In order to explore whether or not host proteins targeted by GCRV are involved in essential infection events, we carried out gene ontology analysis and pathway analysis against the total putative host targets. A total of 48 cellular components were annotated (Fig. 6a), including terms of cell junction, membrane, and macromolecular complex. 460 proteins were annotated to cell junction. Pathway analysis using PANTHER classification system was used to identify the significant pathways involving the pathogeny of GCRV, a total of 36 PANTHER pathways were found successfully overrepresented (*p*-value < 0.05, binomial test). The top 20 significantly overrepresented pathways were shown in Fig. 6b. We observed a significant enrichment in signaling and immune pathways, such as interferon-gamma signaling pathway, VEGF signaling pathway, EGF receptor signaling pathway, B cell activation, T cell activation and interferon-gamma signaling pathway. Interferon-gamma signaling pathway is the most prominent pathway in terms of the significance level and enrichment level.

**Fig. 7:**
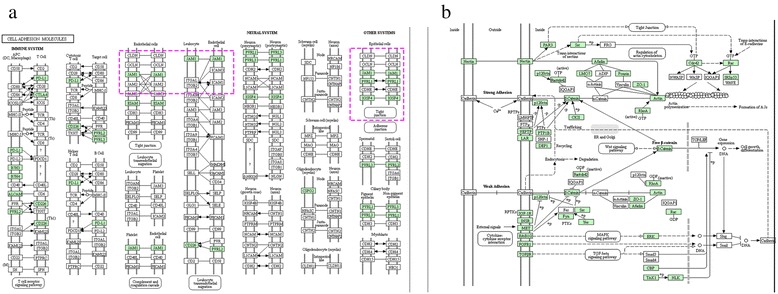
Representative KEGG enrichment pathways of predicted host proteins targeted by GCRV. **a** Cell adhesion pathway, corresponding to PATHWAY Entry: KO04514. **b** Adherents junction pathway, corresponding to PATHWAY Entry: KO04520. Genes inside the red box were putative host proteins targeted by GCRV

Reovirus infection is initiated by attachment of the virus component to different receptors expressed on the cell surface, and spur post binding signaling events, some lead to a cascade of apoptosis, others trigger immune response [38]. However, the events that elicit apoptosis on the cell surface remain unclear. After the KEGG pathway analysis (Additional file 5), we detected two representative pathways related to cell junction, as shown in Fig. 7. Thirteen genes were clustered into the first pathway, named “cell adhesion molecules” (CAMs), corresponding to PATHWAY Entry: KO04514. This pathway involves a large number of proteins expressed on the cell surface and plays a critical role in a wide array of processes, such as hemostasis, immune response, and inflammation. Membrane proteins in this pathway mediate cell-cell interactions that involved in antigen recognition and cellular adhesion. We found JAM genes were widely distributed across several modules of this pathway, suggesting that reovirus attaches to cells via an adhesion-strengthening mechanism by communicating with other molecules. Additionally, 21 other genes were clustered into another pathway, named “adherents junction pathway”, corresponding to PATHWAY Entry: KO04520. Nectins function as cell adhesion molecules (CAMs) to transduce signals through *Cdc42* and *Rac* signaling, indicating that this signaling was also involved in the response to GCRV infection. These findings suggest those genes that expressed on the surface of cell are worthy of further studies and provide more chances for the development of vaccine.

## Discussion

Proteins are the vehicles of immune response and of viral entry into cells, identification of virus-host PPIs is at the heart of virus research. Besides possessing smaller genome and fewer proteins, viruses always distinguish them from other pathogens for lacking of known domains and fast evolutionary rate. Due to high cost of traditional experiments and of transient nature of PPIs between virus and host, identification of virus-host PPIs is a challenge task. With the accumulation of fast-growing sequence and structural data, many computational approaches have been successfully applied to predict pathogen-host interactions [14, 15, 39]. One of the most important strategies is motif-domain interaction based method. Folded, globular domains were once seen as the sole mediators of PPIs. However, accumulating evidence has revealed that the interactome can also be mediated by disordered regions, which natively lack structure, can also be named short linear motifs. Those small motifs also have the trait of evolutionarily plastic to achieve interface mimicry, conferring them the ability to mediate transient interactions and maintain robust cell signaling [40–43]. Recent evidences indicated that motifs may modulate virulence, host tropism, immune escape mechanisms, disease length, and severity of infection. However, this type of interaction has a relatively low affinity due to the limited number of residues [8, 9, 24, 42–45]. Viruses are equipped with high adaptive capacity to battle with their hosts to ensure viral replication, it has been suggested that viruses may employ short, unstructured motifs to mediate interactions with their hosts [38]. Those motifs appear to function in various regulatory interactions by acting as docking sites for certain protein domains, as subcellular-targeting signal, or as recognition sites for protease cleavage (e.g., caspase) [24, 43]. Hence, PPIs mediated by motifs in virus-host system tend to be more transient and regulatory in function. Consequently, the prediction of virus-host interactions from the aspect of structural motif-domain interactions will be an effective approach [42].

Despite that computational approaches have been successfully applied in prediction of PPIs in pathogen-host system, there are still few published reports about PPIs in non-model species conversely. To date, the reports of studies on PPIs of *Aquareovirus*, even *Orthoreovirus*, have been rarely seen. Grass carp hemorrhagic disease, caused by GCRV, has become the most fatal causative agent in grass carp aquaculture. The task of identifying host proteins targeted by GCRV is worthwhile because it may help decipher underlying disease mechanisms and vaccine design. In this study, we adopted a method that based on structural information of motif-domain interactions deposited in two existing data resource, and successfully predicted the PPIs between GCRV and its host grass carp, by using GD108 as the representative GCRV strain. Compared with other computational methods, using structural information as platform to predict PPIs can also provide the detailed information about interfaces that proteins interact through. Although we combined two motif databases to compile a more refined known motif-domain interaction information, the overlapped domain content is limited, suggesting the discrepancy in database construction.

GCRV viruses of different subgroups have not only rather limited sequence identities due to fast evolution, but also the level of pathogenicity, subtype II strains are pathogenic higher and spreading more rapid than subtype I. The cause of this discrepancy may lie in the fact that diversities in protein sequences among different strains lead to different interactome in host. We found that various motifs were identified corresponding to different domains, indicating wide diversity of binding modes for components of GCRV. Motifs of Sigma1-like protein that maybe known as the counterpart of Sigma1 protein in MRV were also detected to interact with JAM proteins in grass carp. Function analysis showed that many proteins are involved in biological regulation and signaling pathways, suggesting that many interactions are transient and partially explain the virus targeted interface tend to be “date”-like [8], that is, they are transiently used by different host targets at different times. The frequently occurring proteins maybe the potential hub genes in the interactome. Pathway enrichment analysis suggests that genes expressed on the surface of cell are worthy of further studies and have more chances for the development of vaccine.

The GCRV infection caused pathology and physiologic dysfunction in a wide range of organs and tissues. Previous study has suggested that reovirus spread from the intestine to sites of secondary infection through bloodstream dissemination [46], indicating that discrepancy in pathogenic pathway among tissues. Consistently, we found that DEGs from different tissues that overlapped with of our predicted interactome displayed different expression patterns on the whole. The expression level in intestinal is higher than that of other three tissues, indicating the functions of these genes are more active in intestinal.

On the whole, it is still difficult to judge the accuracy of predictions for protein interactions in host-virus systems, especially for those viruses such as GCRV that have received less attention than their worldwide burden deserves. It is expected that the findings of our work will contribute to the development of system biology for GCRV infectious diseases, and help guide the identification of novel receptors that GCRV targeted by.

## Conclusion

In brief, we demonstrated the power of motif-based strategy to predict virus-host interactome in a non-model species. Our work provided a systems-based framework for the understanding of the GCRV infectome and diseasome. This is the first draft description of PPIs for GCRV virus-host system, and the results will complement and guide further experiments aiming to identify host hub genes that are necessary for GCRV survival and replication within the host cells. Although the predicted PPIs may contain some false positives, the computational methods provide reasonable amount of interactions which can be further validated by high throughput experiments. Our work will contribute to understanding the mechanism of pathogenesis associated with GCRV infection, and prioritize targets for a rational vaccine-design and disease-resistant breeding.

## Methods

### Data collection and processing

Grass carp protein sequences were downloaded from our previous work [25]. InterProScan software was used for domain annotation using PfamA as the reference database [47]. We retrieved the proteome sequences of GCRV GD108 from Uniprot database [48], as shown in Table 1. In order to build a set of structural descriptors for motif-domain interactions, we collected motif-domain interaction information from two databases, 3did and iELM, respectively [20, 21]. 3did database collects and classifies all structural templates of interactions in the Protein Data Bank, providing molecular details of DDIs and DMIs. The discovery of DMIs requires intensive computation based on structural features, the related method is described in [49]. iELM database is a hub for collecting, classifying and curating information about short linear motifs (SLiMs), the annotated data are manually curated from literature. ELM classes were originally categorized into four different types based on the function of the motif. Motifs in both databases were summarized in the syntax of regular expressions and annotated the corresponding interacting domains that defined from PfamA [47]. Perl script was used to search motif patterns against GD108 protein sequences. Surface accessibilities of motif residues were measured by NetSurfP package [23], if more than half of residues from one motif are predicted to be exposed, then this motif is maintained in the initial interaction database, otherwise it is discarded.

For each motif set of GCRV based on the above two databases, If one GCRV protein was both predicted to interact with the same domain in the two datasets, we considered that this interaction pair was true. However, both databases have different domain contents, which may lead to loss of some important domains. Hence, for both databases, we collected the interactions between database-specific domains and motifs. Furthermore, we evaluated the occurrences of motif patterns, some motif patterns that can be frequently appeared in any protein were discarded (occurrences >4), thus the rarely appeared motifs were reserved and added to the previous interaction pairs. Finally, we linked the motifs to host proteins containing its domain partners.

Previous studies proved that host proteins in virus-host PPIs expressed abundantly across multiple tissues [24], thus we filtered out proteins that rarely expressed in limited tissues (<4) using RNA-seq data from our previous work [25]. Network topology was analyzed by Cytoscape software [26].

### Binding mode analysis of protein interaction

We downloaded the complex structure (PDB ID: 3eoy) between MRV Sigma1 protein and human JAM-A protein from Protein Data Bank (PDB) database [50]. Structure of grass carp JAM-A protein was predicted by using I-TASSER server with default structural template selection [51]. Sigma-1 like protein in GD108 was also modeled with assigning Sigma1 protein (PDB ID, 3eoy) as structural template. Protein docking was carried out by using the Zdock server [52], setting the region around predicted interface between motif and domain as binding sites. Sequence alignments between Sigma1 and Sigma1-like protein were performed by using ClustalW [53]. Besides, structure alignment was carried out in the protocols of Discovery Studio© v2.5.0.9164, built on the SciTegic Enterprise Server platform (Accelrys Software), all the parameters were default values.

### Expression pattern analysis of host proteins targeted by GCRV GD108

Transcriptome data were retrieved from our previous work to investigate the expression pattern of host proteins targeted by virus proteins during various stages of GCRV infection. RNA-seq data were obtained from four diseased grass carp tissues (gill, intestine, liver, and head kidney) with three replicates at four times after (0 h, 1 h, 3 h, and 5 h) GCRV challenge [36, 54]. Expression levels of genes were determined according to the reads per kb per million reads. For each time point (0 h, 1 h, 3 h, and 5 h), we identified DEGs compared with the profile of 0 h time point independently (*p*-value < 0.05, |log2 (Fold_Change)| > 1.5). The four resulting DEGs sets were merged together, and mapped to our predicted host targets. Hence, the overlapping genes were not only host targets but also demonstrating different expression, we analyzed the expression profile of these genes.

### Gene function and pathway enrichment analysis of host proteins targeted by GCRV GD108

Gene functional annotation for host proteins targeted by GCRV GD108 was performed by using Blast2GO software [55]. Pathway enrichment analysis was also carried out using the PANTHER classification system and Cytoscape plugin ClueGO [56, 57]. The KEGG Automatic Annotation Server (KAAS) was used for KEGG orthology assignments and automatically generation of KEGG pathways, BBH (bi-directional best hit) method was adopted to assign orthologues [58].

## Additional files

Additional file 1: Table S1. Information for predicted motifs information of proteins in GCRV GD108 based on 3did database. (XLSX 51 kb)
Additional file 2: Table S2. Information for predicted motifs information of proteins in GCRV GD108 based on iELM database. (XLSX 88 kb)
Additional file 3: Table S3. GCRV virus-host interaction network. (CSV 411 kb)
Additional file 4: Table S4. Information for different expressed genes overlapped with our predicted host proteins. (XLSX 11 kb)
Additional file 5: Table S5. Enriched pathways of predicted host proteins targeted by GCRV. (XLSX 40 kb)

## Abbreviations

DDIs: Domain-domain interactions
DEGs: Differentially expressed genes
DMIs: Domain-motif interactions
GCRV: Grass carp *reovirus*
MRV: Mammalian *reovirus*
PPIs: Protein-protein interactions
